# 3D and 4D Ultrasound: Current Progress and Future Perspectives

**DOI:** 10.1007/s12410-017-9440-2

**Published:** 2017-11-10

**Authors:** Susan H. Kwon, Aasha S. Gopal

**Affiliations:** Research Department, 100 Port Washington Blvd, Roslyn, NY 11576 USA

**Keywords:** 3-dimensional echocardiography, Valvular heart disease, Transcatheter valve replacement, Ventricular function, Strain imaging

## Abstract

**Purpose of Review:**

Three-dimensional (3D) echocardiography (3DE) and 4-dimensional echocardiography (4DE), also known as real-time (RT) 3DE (RT3DE), are rapidly emerging technologies which have made significant impact in the clinical arena over the years. This review will discuss the recent applications of 3DE in diagnosing and treating different types of cardiovascular disease.

**Recent Findings:**

Recent studies using 3DE expanded on prior findings and introduced additional applications to different cardiac conditions. Some studies have used 3D parameters to prognosticate long-term outcomes. Numerous innovative software designs including fully automated algorithms have been introduced to better evaluate valvular heart disease and cardiac function.

**Summary:**

With further evolution of 3DE technologies, this imaging modality will emerge as a powerful tool and likely become the imaging modality of choice in the diagnosis and management of various cardiac disorders.

## Introduction

Significant progress in echocardiography has taken place in the last three decades transitioning from M-mode imaging to 2-dimensional (2D) imaging to the emerging role of 3-dimensional (3D) imaging modality. 2D echocardiography (2DE) has made significant impact on the evaluation of various cardiac diseases, and this modality became the clinical guidelines for management of heart disease and an integral part of routine clinical practice. However, 2DE has several well-known limitations, including lack of 3D spatial coordinates, geometric assumptions, apical foreshortening, and limited boundary recognition. Therefore, it became evident that displaying and understanding the complex 3D structures and physiological systems of the human body would be better accomplished using 3D visualization and analysis.

The early prototypes of 3D echocardiography (3DE) systems used 2D image acquisitions with free-hand scanning where an external 3D spatial coordinate system was provided by an acoustic spatial locator and using either an acoustic or magnetic system of emitters and receivers that were mounted on the transthoracic imaging transducer; images were acquired and reconstructed offline to create 3D data sets [1]. Using the surface rendered reconstructions of the left ventricle (LV), determinations of LV volumes and ejection fraction (EF) were validated to be accurate when compared to several reference modalities, including cardiac magnetic resonance imaging (CMR), cine-ventriculography, and multi-gated radionuclide scintigraphy, as well as found to be superior to conventional 2DE imaging [1–4]. However, the use of 3DE remained limited due to complicated data acquisition techniques and prolonged off-line data analysis. The 3D technology continued to improve with advances in transducer technology. With the introduction of sparse array matrix transducer, 3D volumetric data acquisition was possible, but with suboptimal image quality. Then, the 3D technology took a major turn with the development of the fully sampled matrix array transducer, higher processing 3D echocardiographic systems, and introduction of real-time (RT) 3DE (RT3DE). With the capacity to easily acquire data, visualize RT3D rendered images of the heart on display, and post-process data with software development, 3DE became a widely available tool and increased use of this modality in clinical practice became possible.

In the past 25 years, 3DE has demonstrated to be useful with incremental value or superiority compared to 2DE in various aspects of cardiovascular medicine. This includes accurate measurements of LV volumes, EF, and mass; evaluation of myocardial deformation and regional wall motion analysis; assessment of structure and function of cardiac valves; contribution as a valuable tool pre-, intra-, and post-catheter-based or intra-operative procedures.

## Applications of 3DE

### Quantification of Ventricular Function and Volumes

#### Left Ventricle

Numerous studies have validated that RT3DE derived LVEF and LV volume measurements are accurate and reproducible, when compared to CMR and radionuclide scintigraphy. Furthermore, many studies demonstrated superiority of 3DE over 2DE with improved intra-observer, inter-observer, and test–retest variabilities [5–10]. The limitations of 2D imaging, such as geometric assumptions, apical foreshortening, and image positioning error, do not apply to 3D imaging. However, despite these findings, several studies reported underestimation of LV volumes by RT3DE. This was thought predominantly due to boundary tracing differences due to image quality and depending on the modality chosen as the reference modality. The underestimation of 3DE volumes was significant in subjects with large ventricular size and heart failure [11]. Fully automated endocardial trabecular contouring algorithms have been used and validated to measure volumes and EF compared to CMR in patients in sinus rhythm and in atrial fibrillation. To correct for underestimation of volumes, an automated correction was applied to track the compacted myocardium and mean volume differences between RT3DE and CMR were eliminated. Rapid, accurate, and reproducible EF and LV volumes were obtained by this method for patients in sinus rhythm and atrial fibrillation [12]. Another method of addressing underestimation of RT3DE volumes compared to CMR is use of contrast agents which may improve endocardial border recognition in RT3DE. This may be the case particularly in patients with poor acoustic windows. However, since contrast agents can sometimes cause attenuation and create difficulty in identifying the valve planes, use of contrast agents together with power modulation imaging that uses low mechanical indices provides uniform LV opacification [13].

More recently, studies on 3DE LV chamber function have focused on practicality of this technique and possible integration into routine clinical practice. With 3DE, the limitation is often due to additional time needed to obtain and manually trace the 3D images for LVEF and LV volume calculation as well as lack of 3D experience. Tsang et al. tested the accuracy and feasibility of a novel automated software (HeartModel, Phillips Healthcare) that quantifies LV and left atrial volumes and LVEF using 3D transthoracic echocardiography (TTE) data sets with and without manual contour manipulation that are comparable to conventional manually traced 3D measurements and CMR. They concluded that the novel automated algorithm is feasible, reproducible, and timesaving, and such a technique would be necessary to integrate 3DE LV and LA chamber measurements into clinical practice [14•]. Otani et al. also studied the utility of the same novel fully automated quantification software (HeartModel, Philips Healthcare) with single-beat 3D TTE full volume acquisition in patients with atrial fibrillation. They found that this algorithm reliably and rapidly determines the averaged LV volumes and function during multiple consecutive beats and that this technique may serve as a new way of quantifying LV chamber in patients with atrial fibrillation in routine practice [15].

Newer approach for assessing blood flows within the LV using fully automated software has also been reported. Thavendiranathan et al. used the velocity information encoded in the volume color Doppler data together with simultaneous RT3D TTE imaging of the mitral annulus and the left ventricular outflow tract (LVOT) to quantify mitral inflow and aortic stroke volumes in the same cardiac cycle. There was excellent correlation between these automated measured values with CMR stroke volumes. They also found that this automated technique is rapid, feasible, reproducible, and accurate for measuring mitral inflow and aortic stroke volumes and is superior to manual 2D TTE-based measurements. They concluded that this method may be practical in routine practice to measure stroke volumes, regurgitant volumes, and cardiac shunt fractions [16].

Furthermore, recent studies have also focused on outcome data. Stanton et al. studied 455 patients who underwent LV assessment with 2D and 3D TTE and found that 3D LVEF and 3D LV end systolic volume were the strongest predictors of outcomes (all-cause mortality or cardiac hospitalization) than any of the 2D TTE parameters over a follow-up of 6.6 ± 3.4 years [17•]. Gripari et al. studied 64 patients who underwent percutaneous mitral valve repair (PMVR) and evaluated the short-term (30 days) and mid-term (6 months) LV and right ventricular (RV) chamber remodeling based on volume using 2D and 3D TTE and LV sphericity and conicity indices by 3D TTE. They found that after PMVR, a thorough 2D and 3D TTE analysis of biventricular volume and function as well as LV morphology to assess post-PMVR outcomes is feasible and highly reproducible. In addition to reduction in mitral regurgitation (MR), there was favorable LV remodeling to a more conical shape from an abnormal spherical shape at short- and mid-term follow-up. Furthermore, RV volumes and function post procedure were preserved [18].

##### Strain Analysis

Numerous studies have demonstrated that LV performance can also be assessed by myocardial deformation or strain, which can be performed by a technique known as speckle-tracking echocardiography (STE). This technique uses a pattern-matching algorithm and permits tracking through space and time of all anatomic features including the myocardium present in the echocardiographic image. While 2D STE is limited due to difficulties in tracking the speckles when the speckles move in and out of the 2D imaging plane, 3D STE overcomes this limitation.

Using 3D STE, studies have shown that LV volume assessment is more accurate and reproducible compared to 2D STE [19]. 3D STE also provides new method for analyzing regional function and new 3D parameters to assess LV systolic function such as global area strain (GAS) (percentage of deformation in the LV endocardial surface area defined by the longitudinal and circumferential strain vectors). Recent studies have demonstrated that, on a regional level, GAS is more accurate and reproducible than the other parameters [20]. GAS is strongly correlated with LVEF [21], and as heart function declined from normal to stage D, GAS progressively decreased [22]. In a study of patients with coronary artery disease, 3D area strain is correlated with the severity of transmural extent of necrosis, but 3D strain vector only decreased when necrosis extent was > 75% [23]. More recently, Zhou et al. used a novel fully automated 3D STE algorithm measuring rotational mechanics and found that it is feasible and reproducible compared to tagged CMR and feature-tracking CMR strain assessment [24]. However, while these novel parameters and algorithms of quantifying LV myocardial performance seem promising, proprietory algorithm differences and vendor variability need to be addressed for these indices to be utilized for diagnostic or prognostic consideration [25].

##### Left Ventricular Mass

The Framingham Heart Study as well as other studies have shown that elevated LV mass is a strong, independent predictor of cardiac events and death [26]. Initial studies showed that anatomic in vivo animal validation provided the best results for 3D echo reconstruction compared with the truncated ellipsoid and bullet algorithms [27]. In vivo validation of LV mass by 3DE reconstruction in normal subjects compared to CMR as the reference showed excellent correlation with good inter-observer variability and no statistical difference from CMR values. Furthermore, 3DE reconstruction achieved improved correlation (by twofold to threefold) with CMR over conventional M-mode and 2DE used to compute LV mass [28]. LV mass determination by 3DE reconstruction was also anatomically validated in humans undergoing heart transplantation using the true weight of the left ventricle of the explanted hearts (range 125–433 g) as the reference standard and compared to M-mode Penn, 2DE area length, and truncated ellipsoid algorithms. This study demonstrated that 3DE reconstruction is a highly accurate and reproducible method and that 3DE reconstruction accuracy was fourfold superior to 2DE and ninefold superior to M-mode echocardiography [1]. In addition, RT3DE has shown to be feasible in calculating LV mass. One study demonstrated that RT3DE assessment of LV mass using a single-beat capture method was practical and more accurate than 2D or M-mode in patients with hypertrophic cardiomyopathy [29]. While 3DE has shown to be more accurate than M-mode and 2DE, a meta-analysis of 25 studies including 671 comparisons demonstrated that 3DE still underestimated LV mass compared to CMR [30]. A recent study using 3D TTE for LV mass measurement in healthy subjects compared to CMR showed that this is an accurate method. Between the American and Japanese populations, they observed age and gender dependence without ethnic dependence, and this study provided normal reference values of 3DE derived LV mass indices [31].

##### Left Ventricular Remodeling, Sphericity, and Regional Function

Studies have shown that 3DE reconstruction is useful in determining measures to assess LV remodeling beyond LV mass, including analyzing LV endocardial surface area, infarct subtended surface area, and infarct subtended volume and volume/mass ratio. This is accomplished by calculating a 3D surface area/volume ratio and indexing it to a surface area/volume ratio of a sphere. As the LV undergoes adverse LV remodeling and becomes more globular and spherical, the sphericity index increases [32].

LV volume can be further broken down into 16 or 17 regional segments, and volume in each of these segments can be tracked over the full cardiac cycle to generate time-volume curves. In addition, a bull’s-eye plot can be made of time-volume curves which are generated from the entire LV surface. These time-volume curves and plot can be color-coded to show differential segment contraction (normal segment as blue and late contraction as red). These tools may be useful in evaluating a patient for a biventricular pacemaker. Furthermore, regional volumes assessed in a group of patients were compared against CMR as a reference; agreement levels were very high in basal and mid-ventricular segments, but lower near the apex. This difference may be due to limited endocardial definition near the apex on both 3DE and short axis CMR images affected by partial volume artifacts in this region [33]. RT3DE also provides different ways of evaluating regional LV wall motion which may be applied to interpreting stress echocardiograms. RT3DE can display the entire myocardial volume in a multi-slice panel, allowing a comprehensive assessment of regional wall motion rather than viewing the LV in conventional short axis and apical images where only limited aspects of the myocardium are visualized.

##### Serial Evaluation

Test–retest variability is a measure to assess the utility of a test to assess a parameter in a serial manner. The lower test–retest variability of 3DE compared to 2DE has been well established. In patients undergoing sequential LVEF and volume quantification during cancer chemotherapy, accurate and reproducible quantification of these measures with the lowest temporal variability is vital for clinical decision-making. Marwick et al. found that in patients with stable function while undergoing chemotherapy for breast cancer, non-contrast 3DE was the most reproducible method for LV volume and LVEF measurements over 1 year of follow-up. In addition, 3DE showed the best intra-observer, inter-observer, and test–retest variabilities [34].

##### Post-Myocardial Infarct Risk Stratification

One potential valuable use of 3DE LV quantification is for risk stratification of patients with moderate LV dysfunction post-myocardial infarction (post-MI) or heart failure to guide management with offering lifesaving treatment, i.e., implantable defibrillator placement and/or biventricular pacemaker, based on the post-MI EF. Gopal et al. risk stratified routine methods (2DE, planar multi-gated radionuclide angiography, and cine-ventriculography) compared to 3DE and CMR in patients with myocardial infarction or congestive heart failure and EF ≤ 40%. 3DE had the lowest misclassification rate, stronger correlation, and less bias than 2DE. Based on analysis of variance, 3DE but not 2DE was equivalent to CMR. This study showed that routine methods are inadequate and 3DE is superior for risk stratification by EF post-MI. Furthermore, 3DE with threefold lower test–retest variability than 2DE establishes its use for serial monitoring [35].

#### Right Ventricle

Accurate determination of RV size and function is important for management of various disease processes, including congenital heart disease, pulmonary hypertension, myocardial infarction, heart failure, and pulmonary embolism. Thus, an accurate and easily repeated noninvasive imaging modality for serial evaluation of patients would be essential. However, noninvasive assessment of RV structure and function based on 2D geometric modeling has been challenging due to its complex crescent shape consisting of the inflow tract, the infundibulum, and the apex which are often not all visible simultaneously using 2D imaging techniques. Its retrosternal location also causes suboptimal image quality. In addition, RV myocardial fiber arrangement is different from the LV resulting in peristaltic contractile function. This difference prevents the application of established echocardiographic methods for LV function to assess the RV function [36].

Given the prognostic importance of RV dilatation and function in various disease states, different imaging modalities have attempted to better evaluate the RV. CMR has become the gold standard due to its ability to quantitate RVEF and volumes without concerns of geometric assumptions. Although 2D echocardiographic parameters of RV function, including tricuspid annular plane systolic excursion (TAPSE), basal RV free wall tissue velocity, and fractional area change (FAC), have been established and validated against CMR, technical limitations remain.

Some of the earlier 3D TTE studies showed underestimation of RV volumes and EF compared to CMR [37] or that there was no significant advantage of 3DE compared to 2DE [38]. However, later studies demonstrated that 3DE evaluation of RV volume and EF is accurate, feasible, and reproducible [39]. Recently, novel approaches using new software based on RV-focused 3DE acquisition have been used to better assess the RV and showed that wide range of RV size and function can be estimated accurately and reproducibly [40]. Addetia et al. developed a method to quantify RV shape and tested its ability to differentiate normal subjects from patients with pulmonary hypertension with pressure overloaded RV. Their findings showed that with pulmonary hypertension, curvature analysis using 3DE permitted assessment of RV remodeling quantitatively and may be a method to evaluate disease state by tracking differential changes in regional RV shape [41]. More recently, Nagata et al. found that in 446 patients, 3D TTE-determined RV end diastolic volume, end systolic volume, and RVEF showed good correlations to CMR, and over a 4.1 year follow-up, 3D TTE-determined RVEF was independently associated with cardiac death (RVEF < 35%) and major adverse cardiovascular events (RVEF < 41%) in patients with various cardiovascular disease states [42••].

### Evaluation of Valvular Heart Disease

Echocardiography is the main imaging modality for diagnosing and managing patients with valvular heart disease [43]. Numerous studies have demonstrated that 3DE has incremental value or is superior to 2DE in the evaluation of native and prosthetic valvular heart disease. RT3D transesophageal echocardiography (TEE) with its high spatial resolution allows excellent visualization of the anatomic details of the valve and its surrounding structures in any orientation including the “en-face” view. It also provides valuable quantitative analysis in evaluating severity of valvular conditions. In addition, while 3D TEE is known to have superior anatomic imaging compared to 3D TTE, studies have also shown incremental value of 3D TTE over 2D TTE in some valvular disease processes.

#### Mitral Valve

The mitral valve (MV) apparatus is a complex structure which consists of the leaflets, the saddle-shaped annulus, the chordae tendineae, and the papillary muscles. 3DE has significantly contributed to the anatomic and mechanistic understanding of the MV apparatus in the setting of normal function and dysfunctional pathologic states [44, 45]. Specifically, RT3D TEE has demonstrated to be advantageous in localizing and quantifying the lesion, identifying the mechanisms of mitral regurgitation (MR) (i.e., degenerative, functional, ischemic), quantifying MR severity, contributing to intra- and post-operative evaluation, and decision-making.

Several recent studies of the MV and its apparatus utilizing RT3D TEE have provided additional information to our understanding of its structure and function. Zeng et al. assessed the geometry of the MV with different tethering patterns (asymmetric and symmetric) using RT3D TEE to determine the impact on ischemic mitral regurgitation (IMR) severity based on the tethering patterns. They studied 62 patients with at least moderate IMR and found that (1) there are quantitative differences in MV geometry depending on the tethering patterns, (2) the tethering pattern and the total degree of tethering affects IMR severity, and (3) independent of reduced mitral annular dilatation and higher LV function, asymmetric tethering pattern results in increased severity of MR [45]. Yuan et al. found that RT3D TEE is a valuable tool to rapidly and simply identify mitral valve clefts [46]. Obase et al. focused on the submitral apparatus utilizing RT3D TEE to potentially help guide type(s) of intervention(s) needed with MV repair or percutaneous MV procedures. In one study, they concluded that visualization of the submitral apparatus in transgastric views was feasible and permitted accurate measurement of the chordae tendineae lengths [47]. They also studied the relations of average chordal length and LV size and leaflet surface area in patients with degenerative MR (DMR) and functional MR (FMR) as well as in normal subjects. In the three groups, they found no association between chordal length and LV dimensions; with FMR, there was no elongation of chordal length despite LV enlargement; with DMR, chordal length was elongated and there was positive correlation with leaflet surface area and mitral annular perimeter [48]. Hung et al. showed that if there is papillary muscle displacement outside of the mitral annulus after ring annuloplasty for IMR, MR persists due to persistent abnormal leaflet tethering due to restricted posterior leaflet motion [49]. Lastly, Iwataki et al. studied the effect of extension of calcification to the mitral valve leaflet in patients with aortic stenosis and found that this condition can result in significant non-rheumatic mitral stenosis with decreased leaflet opening and mitral annular area [50].

Furthermore, some groups have utilized novel semi-automated algorithm with RT3D TEE, i.e., anatomical intelligence in ultrasound (AIUS), and concluded that this technique improves accuracy and efficiency in localizing mitral valve prolapse [51]. Aquila et al. utilized a new fully automatic software (eSIE Valves, Autovalve prototype version 1.22, Seimens Medical Solutions, Malvern, PA) to characterize MV/mitral annulus anatomy and function using RT3D TEE in patients with functional MR (FMR) and organic MR (OMR) as well as in control subjects. This software analysis showed that the systolic and diastolic mitral annular sizes are increased for OMR and FMR compared to controls, and there is decreased contractility of the mitral annulus in FMR compared to OMR and controls [52].

With 3DE, color Doppler evaluation as well as anatomic regurgitant orifice area can be assessed and improve the accuracy of diagnosing regurgitant lesion severity. Visualization of the regurgitant jets, including the eccentric jets, is feasible and quantifiable in most patients with RT3D TEE. Also, it has previously been demonstrated that vena contracta area (VCA), effective regurgitant orifice area (EROA), and regurgitant volumes (RV) can be accurately measured with RT3D TEE. Recently, novel approaches and softwares using automatic 3D color Doppler have been utilized to better quantify MR. Heo et al. demonstrated that automatic quantitation of MR using 3D full volume color Doppler TTE was superior to 2D full volume color Doppler TTE, as compared with CMR [53]. In addition, studies in animal models have shown that novel 3D color Doppler ultrasound algorithm based on the field optimization method (FOM) is feasible, accurate, and reproducible in calculating EROA [54, 55].

#### Aortic Valve

The aortic valve is part of the aortic root structure which also consists of the sinus of Valsalva and the inter-leaflet triangles [56]. Numerous studies have demonstrated that RT3D TEE plays an important role in the diagnosis and management of aortic valve disease. RT3D TEE has shown to (1) accurately and reproducibly measure the aortic annular diameter and the distance between the aortic annulus to the coronary ostia with good MDCT correlation; (2) differentiate aortic root geometry in aortic stenosis (tricuspid vs bicuspid); and (3) accurately measure aortic valve area (AVA) and LVOT area by planimetry. Furthermore, prior studies have shown 3DE to be useful in indirect AVA measurement through substituting 3D parameters (planimetered LVOT area and/or 3D derived stroke volume quantification) into the continuity equation to circumvent the pitfalls of 2D AVA estimation based on geometric assumptions [57].

Recently, Caballero et al. showed that many patients with aortic stenosis (AS) have an elliptical and funnel-shaped LVOT which is more prominent further from the annular plane, and measuring 2D LVOT dimension closer to the annular plane best correlates with 3D measurements [58]. Mehrotra et al. compared LVOT geometry and dynamics in patients with AS compared to control subjects. Using 2D TTE and 3D TEE, they showed that in AS patients, the LVOT undergoes remodeling due to increased stiffness and is more elliptical and less distensible at peak systole relative to controls. As a result, greater LVOT cross-sectional area underestimation may result in AS patients compared to control subjects [59•]. Given the anatomic location of the mitral and aortic valve, there is coupling of mitral and aortic valve dynamics where mitral valve disease may affect aortic valve function and vice versa. In one study, severe AS patients with calcification in the aortic-mitral fibrous continuity showed no improvement in MV structure after transcatheter aortic valve replacement (TAVR) due to the calcification [60].

While numerous studies have assessed the role of 2D STE imaging in aortic stenosis, limited number of 3D STE studies has been performed. In a recent study with 104 asymptomatic severe AS patients with preserved LVEF, 3D global longitudinal strain (GLS) was the strongest predictor for future adverse cardiac events over a follow-up of 373 days [61].

#### Tricuspid Valve

3DE has shown to have incremental value over 2DE in the assessment of the tricuspid valve (TV) structure and function. It is well established that the standard 2D views of the TV (right ventricular inflow, parasternal short axis, apical four chamber, and RV focused view) can only visualize two of the three TV leaflets. Furthermore, there is significant variability as to which leaflets are being seen in these views. Addetia et al., using 3D multiplanar reconstruction method, found nonstandard 2D views which can better identify the TV leaflets [62].

In the setting of pacemaker leads, 3DE is useful in identifying the position of the lead as it crosses the TV and improves the assessment of tricuspid regurgitation (TR). Addetia et al. studied the impact of implantable transvenous device lead location on the severity of TR and found that 3D TTE is superior to 2D TTE in identifying lead-leaflet interference which results in TR after device lead placement. This finding suggests 3D TTE should be considered in the evaluation for possible lead interference in patients with significant TR after lead placement. Furthermore, TR quantification using 3D color Doppler VCA is feasible and shows good correlation with 2D methods [63]. For functional TR, RT3D TEE has shown to have incremental value over 2DE in understanding the pathophysiology of TR in patients with atrial fibrillation, where increased TV annular area was significantly associated with TR severity [64].

#### Prosthetic Valves

RT3D TEE is known to accurately evaluate prosthetic valve function with better visualization of the prosthetic valve anatomy as well as potential paravalvular regurgitation or vegetation. Thus, this modality plays an important role during transcatheter interventions for treatment of paravalvular regurgitation [65] or bioprosthesis malfunction [66, 67]. For endocarditis evaluation, RT3D TEE “en-face” view of the prosthesis is very useful in visualizing vegetations and valve dehiscence as well as localizing the regurgitant jets.

### Guidance of Catheter-Based Interventions

With rapidly expanding catheter-based procedures for treatment of structural heart disease, RT3D TEE is known to be a powerful imaging modality for pre-procedural evaluation, intra-procedural guidance, and post-procedural assessment. During the catheter-based procedures, the main advantages of RT3D TEE are the ability to visualize (1) in real time the structure and pathology undergoing intervention in “en-face” view, which is not possible with other imaging modalities, and (2) the intra-cardiac catheters, including the tips and the devices or balloon that are carried, as well as their position relative to the surrounding structures [68].

Since the acceptance of TAVR as an alternative to surgical treatment, this technology has been rapidly expanding and the value of RT3D TEE became well established with this procedure. Recent studies have shown that RT3D TEE plays a vital role in determining the size of the transcatheter valve [69, 70]. Furthermore, RT3D TEE has proven to provide annular measurements that are comparable to computed tomography [71]. Lastly, in addition to guiding the TAVR procedure with high spatial resolution anatomic views, after the procedure, RT3D TEE can be used to determine the location and degree of paravalvular regurgitation [72] as well as used for cardiac function, new wall motion abnormalities, and pericardial effusion.

**Fig. 1:**
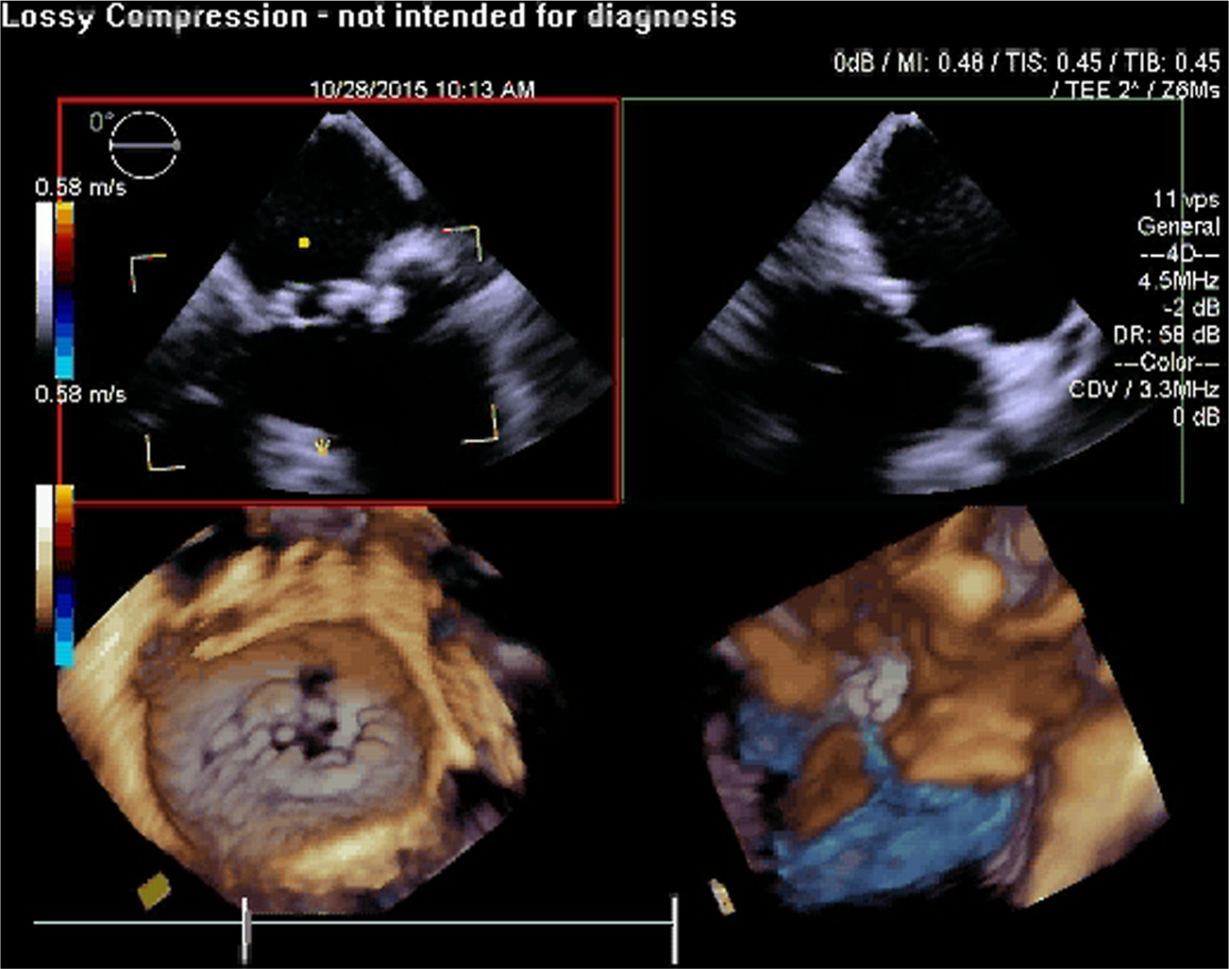
Pre-procedure 3D TEE showing a mitral annuloplasty ring with an unrepaired cleft in the anterior mitral leaflet

**Fig. 2:**
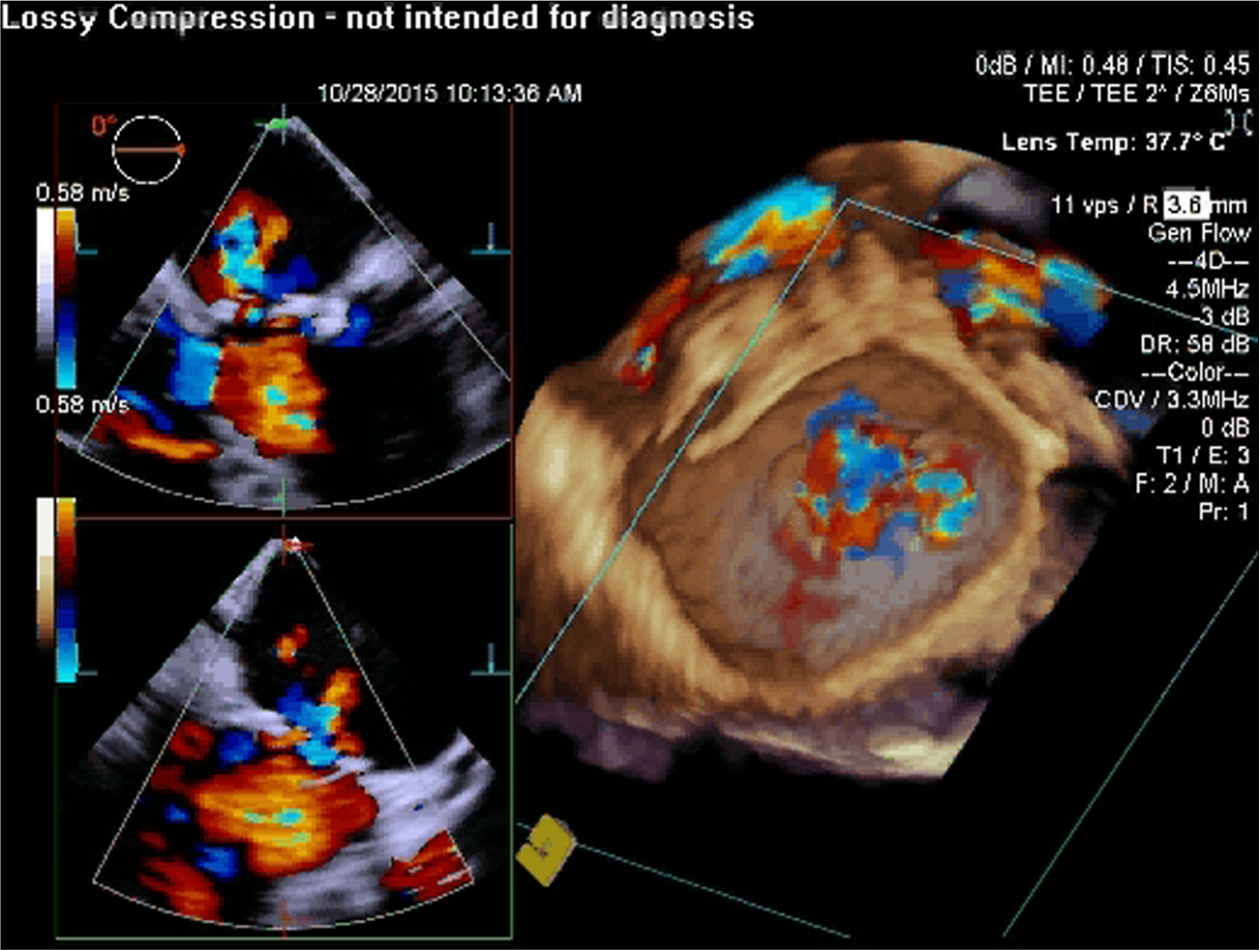
Pre-procedure 3D TEE with color flow Doppler showing severe mitral regurgitation originating from the unrepaired cleft in the anterior mitral leaflet

**Fig. 3:**
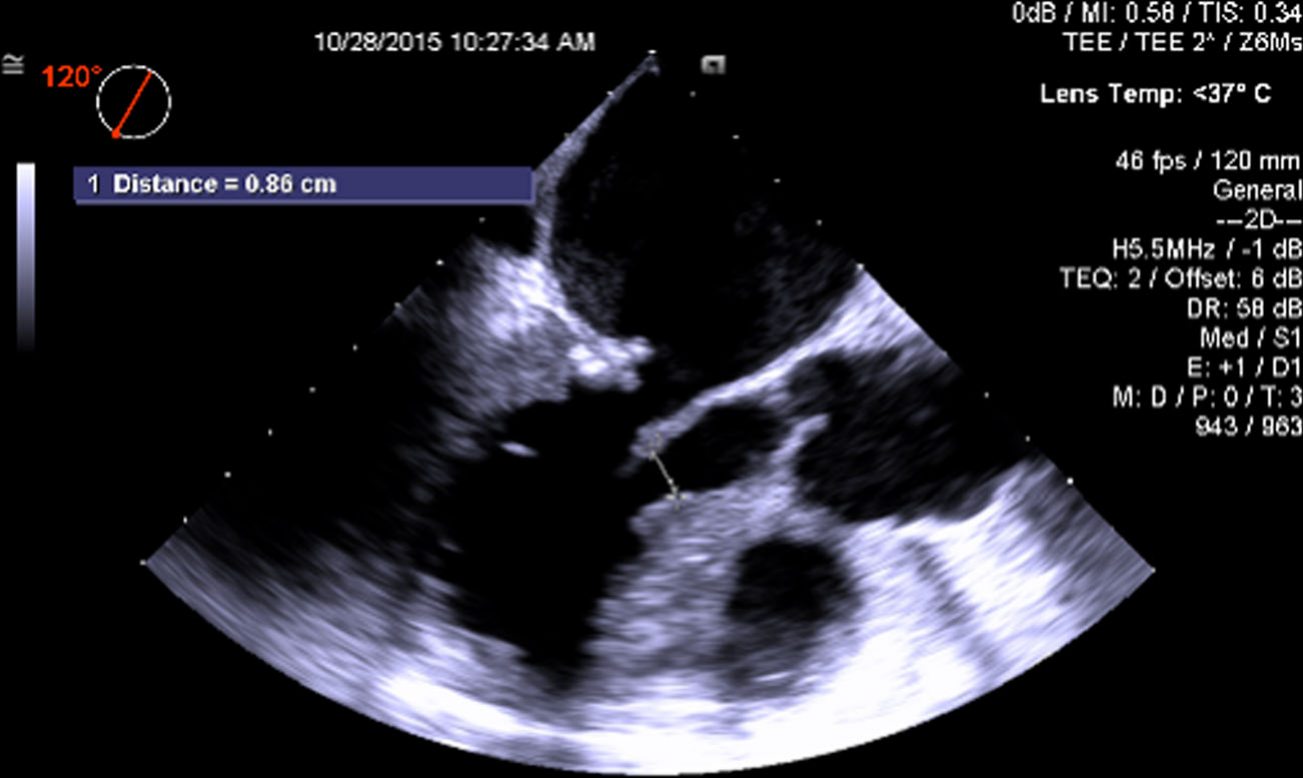
Pre-procedure TEE measurement of the distance between the tip of the anterior mitral leaflet and the septum (important in the assessment of the probability of obstruction of the left ventricular outflow tract from a contemplated transcatheter mitral valve implant)

**Fig. 4:**
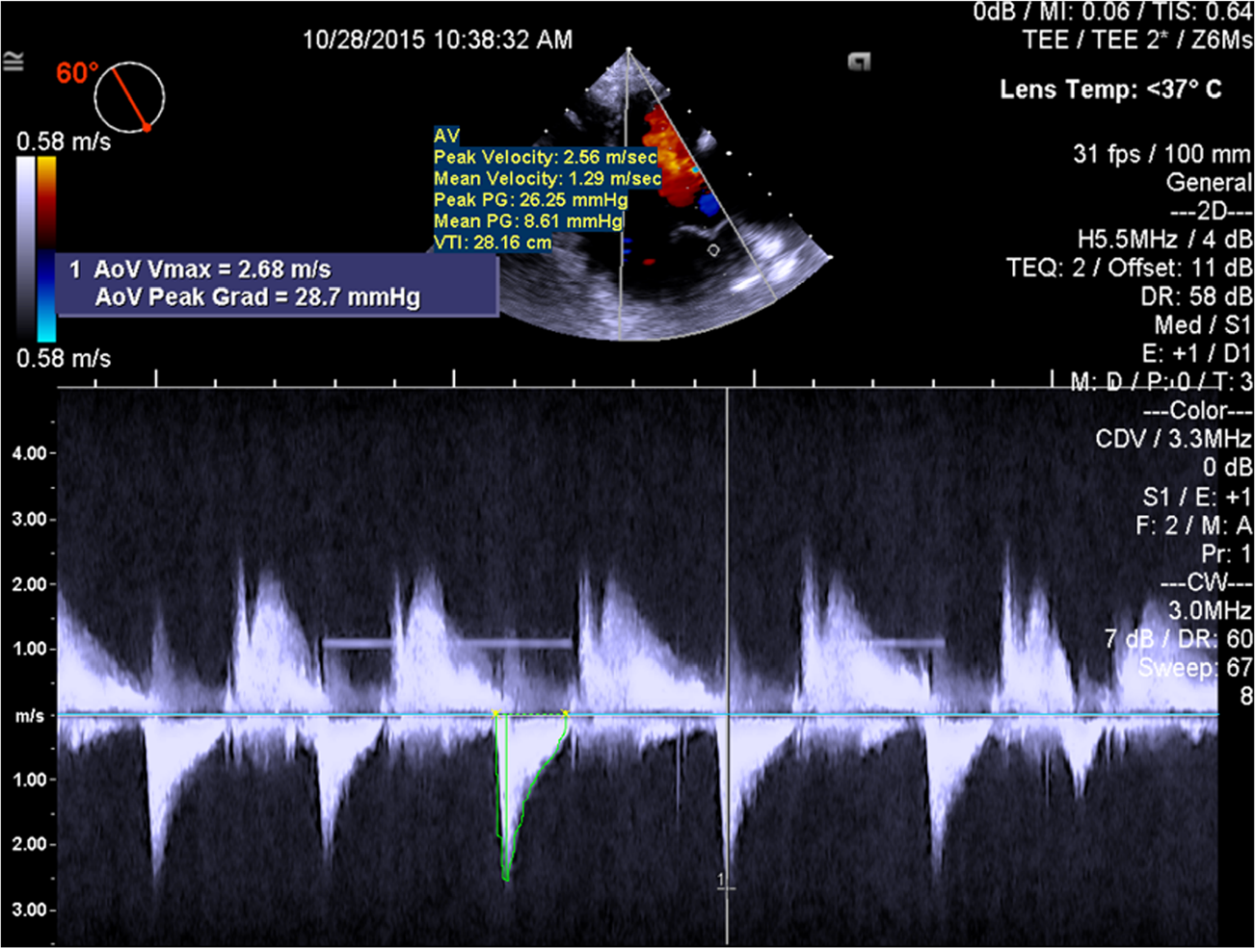
Pre-procedure TEE measurement of the peak and mean gradients across the aortic valve (important to document prior to transcatheter mitral valve implantation to ensure that there has been no obstruction of the left ventricular outflow tract)

**Fig. 5:**
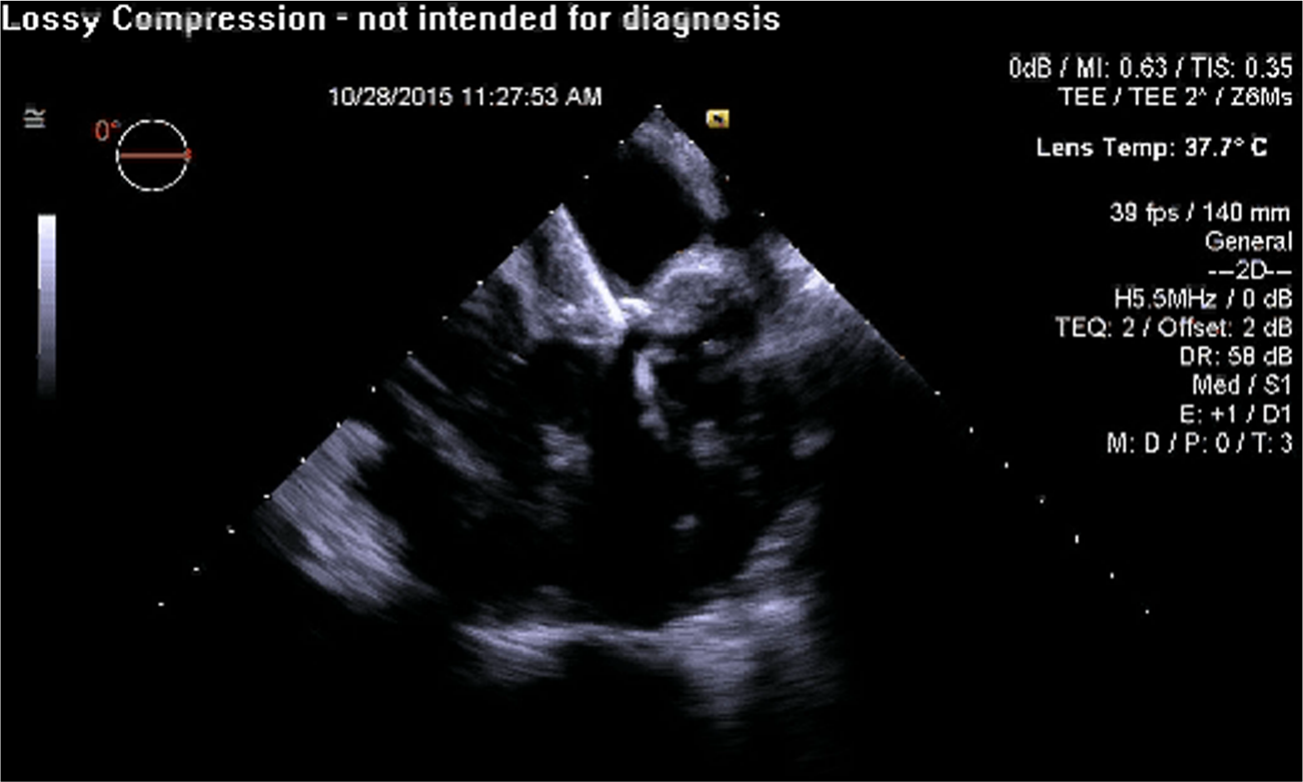
Guidance of the transapical catheter along the true long axis of the left ventricle (important to obtain views of the left ventricle that are not foreshortened)

**Fig. 6:**
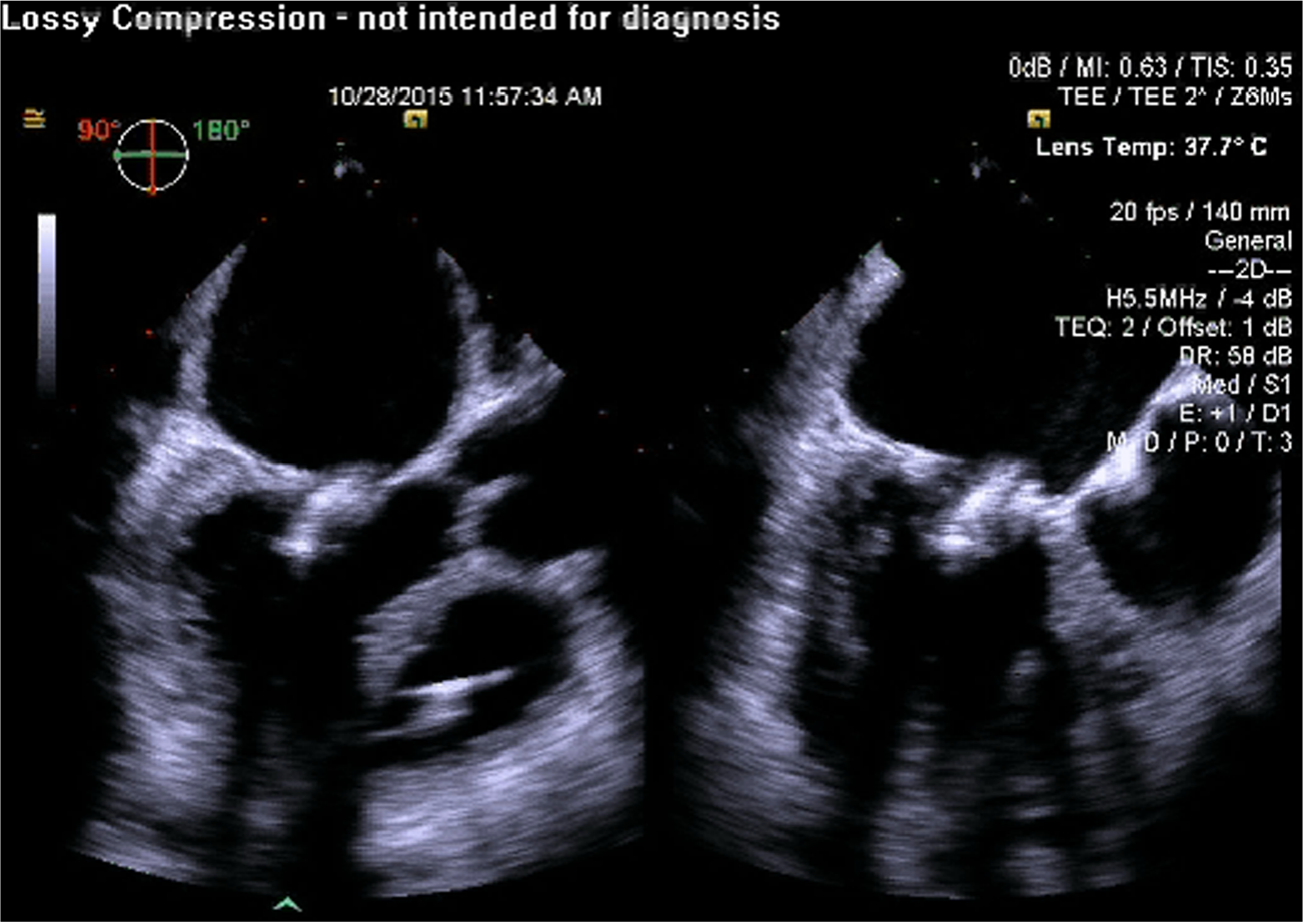
Guidance of the transapical catheter such that is visualized to be in the center of the mitral annulus in the biplane view

**Fig. 7:**
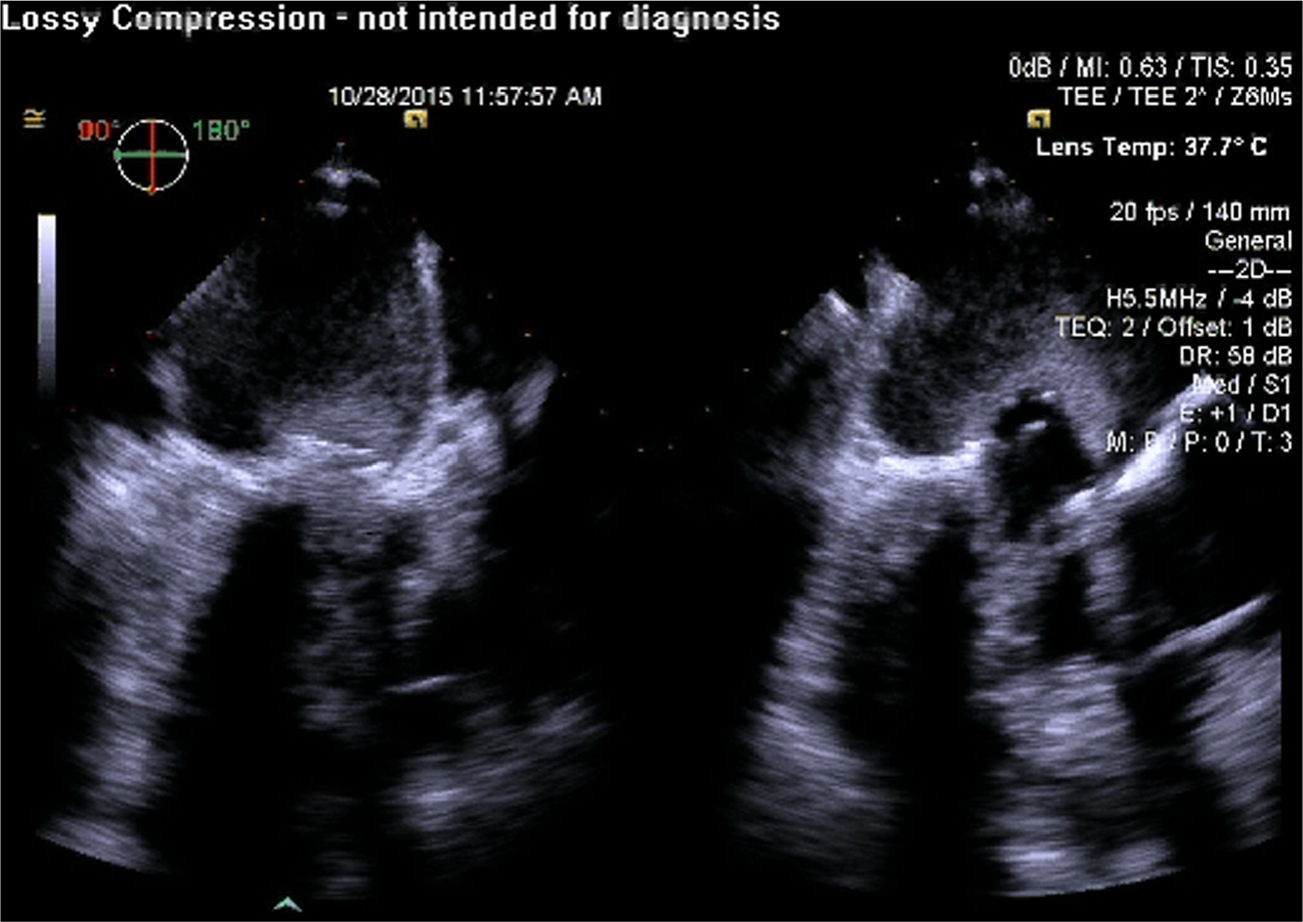
Rapid pacing prior to deployment of the transcatheter valve in order to minimize cardiac motion

**Fig. 8:**
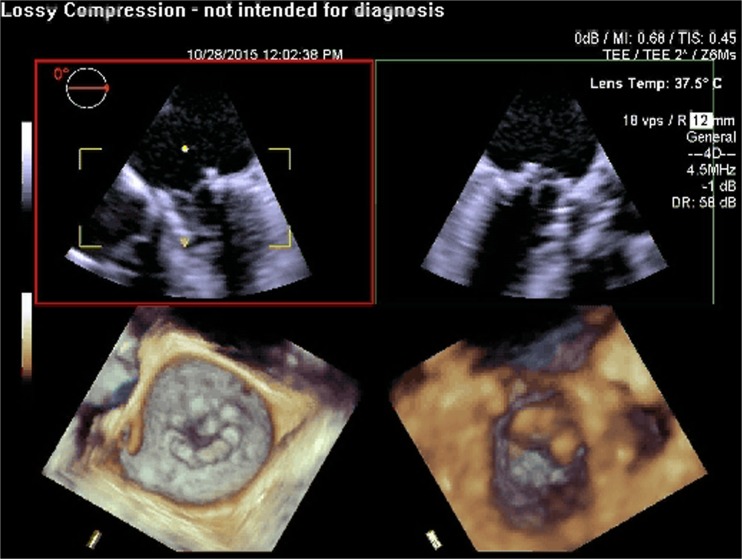
Post-procedure 3D TEE showing a well seated Sapien aortic prosthesis in the mitral position (off label use)

**Fig. 9:**
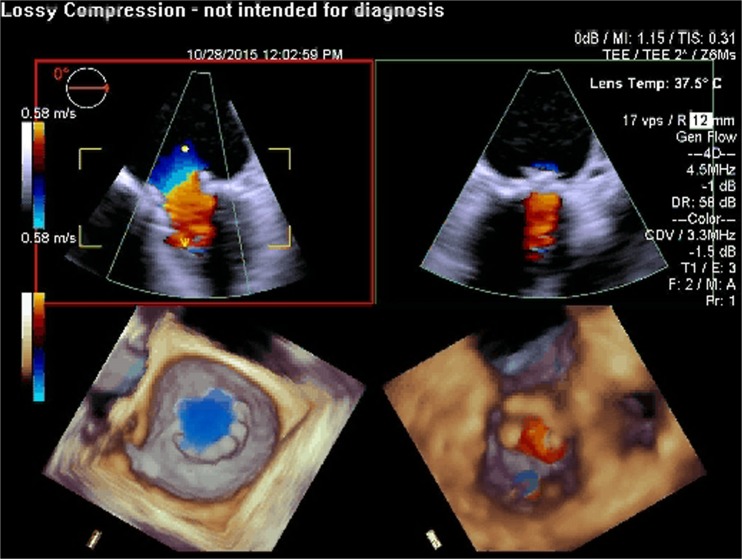
Post-procedure 3D TEE with color flow Doppler showing no significant residual mitral regurgitaton after deployment of a Sapien aortic prosthesis in the mitral position using a transapical approach (off label use)

RT3D TEE has also shown to play an important role with MitraClip implantation [73]. Comparing RT3D TEE and 2D TEE in percutaneous mitral valve repair, 3D imaging is more effective in optimizing the trans-septal puncture site, guiding and positioning the clip delivery system, positioning the regurgitant jet, visualizing the clip with respect to the valve orifice, and evaluating the residual regurgitant jet(s) [74]. Furthermore, the ability of RT3D TEE to view the MV in an “en-face” view plays a vital role as this allows a thorough assessment of the valve anatomy as well as the regurgitant jets in terms of location, number, and severity. RT3D TEE will play an important role in the development of transcatheter mitral valve implantation procedures both from percutaneous transfemoral and from transapical approaches (Figs. 1, 2, 3, 4, 5, 6, 7, 8, and 9 illustrate the key steps in the guidance of a procedure of this kind).

### Future Developments/Direction

In order to enhance 3DE applications into routine clinical practice, further improvement in transducer technology as well as software and machinery development are warranted. Especially with 3D TTE, the improvement of temporal and spatial resolution would significantly expand its utility for various disease processes and integrate routine 3D imaging as part of 2D TTE imaging. Improvement in 3D color Doppler technology as well as software development will permit novel ways to analyze structure and function. In addition, standardization of various platforms and algorithms among vendors is necessary in order to develop long-term RT3DE prognostic parameters. Lastly, use of fusion imaging incorporating RT3DE with other modalities may provide incremental value in diagnosis and treatment of various cardiac diseases.

## Conclusions

The field of 3DE has made tremendous progress since its introduction three decades ago. Recently, RT3D TEE has demonstrated to play a vital role particularly in the diagnosis and management of valvular heart disease. With further advancement of the 3D technology with improvement in transducer technology, image quality, standardization, and software development, it is anticipated that 3DE will progressively become more integrated into the clinical guidelines and routine practice.
